# MonotomidGen – A matrix-based interactive key to the New World genera of Monotomidae (Coleoptera, Cucujoidea)

**DOI:** 10.3897/zookeys.634.9857

**Published:** 2016-11-21

**Authors:** Thomas C. McElrath, Olivia F. Boyd, Joseph V. McHugh

**Affiliations:** 1 Department of Entomology, University of Georgia, 455 Biological Sciences Athens, GA 30602, USA; 2 Department of Integrative Biology, Oregon State University, 3029 Cordley Hall Corvallis, OR 97331, USA

**Keywords:** interactive key, data matrix, identification, morphology, LUCID, minute clubbed beetles

## Abstract

A matrix-based Lucid^TM^ key is presented for the twelve genera of Monotomidae (Coleoptera: Cucujoidea) represented in the New World. A general overview is given for the features and technical specifications of an original interactive key for the identification of these genera. The list of terminal taxa included with the key provides a current summary of monotomid generic diversity for the Nearctic and Neotropical regions.

## Introduction

Matrix-based (also known as interactive, multi-access, multi-entry, or filter-style) keys offer vast advantages over traditional dichotomous identification keys. Some advantages include: freedom to follow more than a single path, ability to use only subsets of characters, integration of non-traditional (e.g., biology, distribution) and overlapping characters, effective use of multi-state characters, and inclusion of numerous graphics (Penev et al. 2009, 2012). These keys have been used successfully to overcome the challenges of identification of many groups of organisms, including various flies (Lyons and Dikow 2010; Ceretti et al. 2012), thrips (Mound et al. 2012), aphids (Favret and Miller 2012), and beetles (Lawrence et al. 2010; Lord et al. 2011; Nearns et al. 2016). However, many other challenging groups could use such powerful identification tools.

With 117 described species in twelve genera, the New World Monotomidae (also known as the “minute clubbed beetles”) are a small group of mostly mycophagous and predaceous beetles within the superfamily Cucujoidea. The worldwide generic diversity was last reviewed by Sen Gupta (1988). Since that review, nine new genera have been described, including one from the New World (Pakaluk and Ślipiński 1993; Pal 1996; Sen Gupta and Pal 1995). The North American genera were briefly reviewed and an identification key was provided by Bousquet (2002a). Many Nearctic genera have been reviewed relatively recently (Bousquet 1990, 2002b, 2003a, b, c; Bousquet and Laplante 1999). Despite these reviews, identification remains difficult, especially to non-specialists who are unfamiliar with the diagnostic characters. Since Monotomidae are important components of forest ecosystems as predators of scolytine bark beetles (Gregoire et al. 1985), vectors of fungal pathogens in trees (Hinds 1972), and pollinators (Jenkins et al. 2015), non-specialists frequently encounter them and need to make confident determinations. In addition, some monotomids, such as *Rhizophagus parallelocollis*, *Monotoma longicollis*, *Monotoma spinicollis*, *Monotoma johnsoni*, *Monotoma picipes*, and others (Kuschel 1979; Bousquet 1990; Bousquet and Laplante 1999; Jelinek 2007), are being spread worldwide through human commerce or expanding their native range (Peck and Thomas 1998), and their effects on ecosystems will remain undocumented until they are identified. However, monotomid identifications have been complicated by the inaccessibility of taxonomic literature, lack of a recent, synthetic, genus-level treatment, and inadequacy of available graphics (habitus photographs, electron micrographs, and illustrations) to interpret many diagnostic features.

To address these issues, an interactive matrix-based identification key was developed for the twelve described genera of New World Monotomidae. This key is based on a matrix of 46 characters derived from morphometrics, discrete anatomical features, distributional data, and ecology. Included are illustrations of diagnostic features and dorsal and ventral photomicrographs of reliably determined representatives of each genus. Complete taxonomic coverage was possible for some genera, allowing inclusion of photomicrographs and morphometric data for all known species.

## Project description

### Taxonomic coverage

This key covers 12 of the 12 genera belonging to the family Monotomidae that are currently known to occur in the New World (Bousquet 2009).

### List of the terminal taxa included in the current version of the identification key (last update November 2016)

***Aneurops*** Sharp, 1900; ***Bactridium*** LeConte, 1861; ***Crowsonius*** Pakaluk & Ślipiński, 1993; ***Europs*** Wollaston, 1854; ***Hesperobaenus*** LeConte, 1861; ***Leptipsius*** Casey, 1916; ***Macreurops*** Casey, 1916; ***Monotoma*** Herbst, 1793; ***Phyconomus*** LeConte, 1861; ***Pycnotomina*** Casey, 1916; ***Rhizophagus*** Herbst, 1793; ***Thione*** Sharp, 1899.

### Photomicrographs of terminal taxa

Each genus included in the key has at least one associated dorsal and ventral photomicrograph. For most genera, multiple photomicrographs were provided in order to illustrate the range of intrageneric diversity. All photomicrographs represent either type specimens, authoritatively identified museum material, or material determined by the first author (TCM). Illustrative shots of important characters are provided within the key, and larger dorsal and ventral habitus photographs are included within the Fact Sheets section of the website.

### Characters used in the key

#### General features

Characters used for identification were derived from existing literature (Sen Gupta 1988; Bousquet 2009) but then confirmed and scored from specimens in the University of Georgia Collection of Arthropods (UGCA) and the Smithsonian Institution National Museum of Natural History (NMNH). Anatomical terminology follows that of Bousquet (2009) and Sen Gupta (1988), the most comprehensive morphological treatments of Monotomidae to date.

The data matrix forming the foundation for this key is based on 46 anatomical, distributional, and ecological characters. These features are encoded into characters with a range of two to eight possible states. Most characters refer to external anatomical features of the adult form that are easily visible without preparation or dissection. Because multi-access keys provide users with greater flexibility than dichotomous keys, hard-to-view and rarely available features also are included. For example, ecological characters are provided for unusual cases when such information is available. The key includes several hind wing characters, usually visible only after dissection and preparation, because they are very valuable for separating genera. In addition, five morphometric characters are included. The diagnostic range values for these characters were based on measurements of multiple species within each genus, including measurements from as many reliably identified specimens as was reasonable. By measuring many diverse individuals representing each genus, more accurate estimates of the range in sizes was possible for these morphometric characters. The morphological characters are sorted by body part (head, mouthparts, thorax, scutellum, legs, hind wing, and abdomen; available via the “Subsets” button on the Lucid Player control bar, and sorted by default) allowing the user to easily focus on particular regions or preparations of a specimen. To quickly narrow some identifications, distributional characters are included.

Most characters are accompanied by supporting images and clarifying explanations within the key, as well as in the “Glossary of Terms” section (see below) of the website.

#### List of the characters used in the key

GENERAL: length (mm); ratio of body length: greatest body width; body shape (lateral view); dorsal surface of the body (setation); elytral color; biology (habitat, known host associations, etc.); geographic distribution

HEAD: ratio of head length: greatest head width (including eyes); head constriction (presence/absence); ratio of temple length: longitudinal length of eye; antennal cavity on ventral side of head (presence/absence); eyes (number of facets); antennal club (number of apparent segments); antennal club (whether distinct)

MOUTHPARTS: maxillary palps (size of second segment); labial palps (size of second segment); mandibular dentation (number of teeth); mandibular cavity (presence/absence)

THORAX: ratio of pronotal length along midline: greatest pronotal width; pronotal disc (vestiture); pronotal disc (shape); lateral margin of pronotum (smooth/crenulate); pronotal disc (impressions); pronotal microsculpture (presence/absence); pronotal puncture density (center of disc only); anterior angles of pronotum (whether projecting); procoxal cavities externally (shape); procoxal cavities (degree of separation); procoxal trochantins (exposure); scutellar microsculpture (presence/absence); scutellar setation (presence/absence); mesocoxal cavities (degree of separation)

LEGS: number of metatarsomeres of male

WINGS: elytral setigerous punctures (arrangement); setigerous punctures on epipleural fold (number of rows); hind wing (presence/absence); number of anal veins; r-m cross (degree of development); subcubital fleck (presence/absence)

ABDOMEN: intercoxal process of abdominal ventrite (shape); metacoxal bead or femoral line (presence/absence); metacoxal bead or femoral line (length of production); first abdominal ventrite of male (special modifications); puncture rows on abdominal ventrites two-four (presence/absence); number of rows of punctures on abdominal ventrites two-four; punctures on abdominal ventrites two-four (size/shape)

## Software technical specifications

Application: Lucid Builder 3.5 (available at www.lucidcentral.org, see website for exact technical specifications and features list)

Key version: 1.0

Requirements for use: Java-enabled browser and internet connectivity

License for use of the key: Creative Commons Attribution License (CC-BY 4.0), which permits unrestricted use, distribution, and reproduction in any medium, provided the original author and source are credited

Web location: http://www.monotomidae.com/MonotomidGen.html

## Data resources

The data underpinning the Lucid Key (Lucid Key files) reported in this paper are deposited in the Dryad Data Repository at http://dx.doi.org/10.5061/dryad.q9p4j

## Website features

### Genus fact sheets

http://monotomidae.com/facts.html

Each of the twelve genera represented in the key are treated and figured with dorsal and ventral habitus images. For each genus, informational sections about the following subjects are provided to assist identifications: Taxonomy, Diagnosis, Biology, Distribution, List of Species (photographed and not-photographed), and Suggested References.

### Resources

http://monotomidae.com/resources.html

An anatomical atlas, glossary of terms, and guide to diagnosing the beetle family Monotomidae are included here. The anatomical atlas illustrates many of the characters used in the identification key and includes an illustration of the dorsal and ventral habitus of *Monotoma producta*, as well as a wing illustration of *Rhizophagus sayi*. The glossary of terms (http://monotomidae.com/glossary.html) provides clarifiying definitions and explanations of all terms included in the interactive key, listed alphabetically, drawn from Nichols (1989), Lawrence et al. (2011), and Nearns et al. (2016). The diagnosis page (http://monotomidae.com/whatis.html) discusses characters that could diagnose a beetle as belonging to Monotomidae. It also provides photographs of taxa that are commonly misidentified as Monotomidae.

### References


http://monotomidae.com/references.html


A list of useful monotomid references is given. Links are provided to available PDFs or websites of these references when not in violation of copyright restrictions.

## Conclusions and future work

During development of this identification resource, several problems became apparent. First and foremost, nearly all monotomid genera included herein require modest or extensive taxonomic work. For the Nearctic region, the problem is not as serious because most genera, with the exception of *Bactridium*, have been at least partially treated within the last 25 years (e.g., Bousquet 1990, 2002a, 2002b, 2003a, 2003b, 2003c; Bousquet and Laplante 1999). *Bactridium* requires extensive work and is currently undergoing revision by TCM. In addition, most other genera represented in the Nearctic harbor some undescribed species (e.g., *Monotoma*, *Aneurops*, and *Rhizophagus*). As new types of data are examined, some currently recognized polymorphic species may be recognized as species complexes. The Neotropical fauna has been far less studied; numerous undescribed species and potentially even genera exist. Species identification in this region almost always requires comparison with type material. Even genus-level identifications of Neotropical specimens should be confirmed by a specialist, though this key will narrow down options for tentative determinations considerably.

Second, the relationships between monotomid genera are poorly understood. No phylogenetic analyses of any kind have been performed for this family. Thus, some morphological characters currently used to delimit genera require investigation to test their success in characterizing monophyletic groups. Some monotypic North America genera (e.g., *Pycnotomina*, *Macreurops*, and *Phyconomus*) should especially be targeted, as they may represent highly autapomorphic lineages nested within other genus-level clades.

Pending completion of a number of alpha taxonomic studies and phylogenetic analyses of the family, it will be possible to update this key to include species as the terminal units, and to more rigorously define the genera, as supported by additional characters. In the meantime, the numerous habitus images and illustrations should provide enough resources for confident genus-group determinations, and the other resources provided within MonotomidGen should facilitate approximate species identification.

This key provides a flexible, powerful, and media-rich information resource for any scientist or non-professional who needs to identify monotomid beetles. In addition, it provides a framework upon which to build future identification resources for this family. Eventually, a worldwide resource for identification of monotomid beetles should be completed to identify the species being transported around the world through human activities. This will allow for quicker identifications and therefore, quicker documentation of the spread of newly adventive species. Taxonomic resources of broader scope such as MonotomidGen can assist those tasked with discovering and identifying these anthropogenic species introductions.
